# Hyperinsulinemic hypoglycemia in children and adolescents: Recent advances in understanding of pathophysiology and management

**DOI:** 10.1007/s11154-020-09548-7

**Published:** 2020-03-17

**Authors:** Maria Gϋemes, Sofia Asim Rahman, Ritika R. Kapoor, Sarah Flanagan, Jayne A. L. Houghton, Shivani Misra, Nick Oliver, Mehul Tulsidas Dattani, Pratik Shah

**Affiliations:** 1 Genetics and Genomic Medicine Programme, UCL Great Ormond Street Institute of Child Health, Great Ormond Street, London, WC1N 3JH UK; 2 Department of Pediatric Endocrinology, Great Ormond Street Hospital for Children, London, UK; 3 Endocrinology Service, Hospital Infantil Universitario Niño Jesús, Madrid, Spain; 4 Pediatric Diabetes and Endocrinology, King’s College Hospital NHS Trust, Denmark Hill, London, UK; 5 Institute of Biomedical and Clinical Science, University of Exeter Medical School, Exeter, UK; 6 Royal Devon and Exeter Foundation Trust, Exeter, UK; 7 Department of Diabetes, Endocrinology and Metabolic Medicine, Faculty of Medicine, Imperial College Healthcare NHS Trust, London, UK

**Keywords:** Hyperinsulinism, Hypoglycemia, Sirolimus, Lanreotide, 18F-DOPA-PET, Transition to adult services

## Abstract

Hyperinsulinemic hypoglycemia (HH) is characterized by unregulated insulin release, leading to persistently low blood glucose concentrations with lack of alternative fuels, which increases the risk of neurological damage in these patients. It is the most common cause of persistent and recurrent hypoglycemia in the neonatal period. HH may be primary, Congenital HH (CHH), when it is associated with variants in a number of genes implicated in pancreatic development and function. Alterations in fifteen genes have been recognized to date, being some of the most recently identified mutations in genes *HK1, PGM1, PMM2, CACNA1D, FOXA2* and *EIF2S3*. Alternatively, HH can be secondary when associated with syndromes, intra-uterine growth restriction, maternal diabetes, birth asphyxia, following gastrointestinal surgery, amongst other causes. CHH can be histologically characterized into three groups: diffuse, focal or atypical. Diffuse and focal forms can be determined by scanning using fluorine-18 dihydroxyphenylalanine-positron emission tomography. Newer and improved isotopes are currently in development to provide increased diagnostic accuracy in identifying lesions and performing successful surgical resection with the ultimate aim of curing the condition. Rapid diagnostics and innovative methods of management, including a wider range of treatment options, have resulted in a reduction in co-morbidities associated with HH with improved quality of life and long-term outcomes. Potential future developments in the management of this condition as well as pathways to transition of the care of these highly vulnerable children into adulthood will also be discussed.

## Introduction

Glucose is one of the principal energy substrates, providing half of the body’s total energy requirements. As the brain can neither synthesize nor store more than a few minutes supply of glucose, its function is solely dependent on maintenance of normal glucose concentrations in the circulation. An abnormally reduced concentration of glucose in the blood is referred to as hypoglycemia. It is a medical emergency and can lead to symptoms due to neuroglycopenia [1].

In healthy individuals, maintenance of a normal plasma glucose concentration relies on a tightly controlled balance between glucose production (dietary intake, glycogenolysis, gluconeogenesis) and its utilization by the tissues (glycolysis, glycogenesis, conversion to fatty acids). A normal endocrine system is essential for integrating and modulating substrate mobilization, interconversion, and utilization. In addition, the endocrine system interacts with metabolic pathways that rely critically on functionally intact enzymes. There are two types of metabolic hormones affecting blood glucose concentrations – an anabolic hormone (insulin), which decreases blood glucose, and several catabolic hormones (such as glucagon, cortisol and catecholamines) which increase blood glucose concentrations.

Hyperinsulinemic hypoglycemia (HH) is the commonest cause of persistent hypoglycemia in infants and children [2] and it can be transient –associated to risk factors- or permanent –linked to genetic mutations-. The risk of permanent brain injury in infants with HH continues to be as high as 25–50% due to delays in diagnosis and inadequate treatment. Despite advances in genetics, improved modes of investigation, novel management options and abrigding pediatric and adult follow-up in holistic multidisciplinary transition clinics, significant morbidity and mortality is still a major issue in children and young adults with HH [3–5].

The present review has been written using a comprehensive and up-to-date literature search on congenital hyperinsulinism/HH including the latest publications available in PubMed (last search in August 2019). It also incorporates clinical and laboratory experience from reference centers for the diagnosis and management of HH, as well as available data from on-going pharmaceutical trials.

## normal blood glucose and hypoglycemia

### Definition of normal blood glucose

Blood glucose concentrations of normal term neonates appropriate for gestational age may range between 1.4–6.2 mmol/l (25–112 mg/dl) during the first 72 h of life; however after that, healthy children and adults will maintain blood glucose concentrations between 3.5–5.5 mmol/l (63–99 mg/dl) [6]. It is difficult to numerically define hypoglycemia given that a single cut-off value cannot suit all individuals in every situation. Therefore operational thresholds are recommended which indicate that in any baby with clinical signs of hypoglycemia, blood glucose levels must be maintained over 2.6 mmol/l (47 mg/dl) except for suspected cases of hyperinsulinemic hypoglycemia in which 3.5 mmol/l (63 mg/dl) should be the cut-off point [7]. However, the Pediatric Endocrinology Society recommends that when a congenital disorder causing hypoglycemia is suspected in a neonate and when confirmed in older infants and children, the aim is to keep plasma glucose concentrations over 3.9 mmol/l (70 mg/dl) [7].

### Causes of hypoglycemia

For hypoglycemia to occur, the rate of appearance of glucose into the plasma space must be less than its rate of utilization [8]. This can be due to defective glucose production, increased glucose utilization, or some combination of the two. Excessive glucose utilization due to hyperinsulinism (exogenous/endogenous) is one of the commonest causes of hypoglycemia. Hypoglycemia can also occur due to deficiencies of various counter regulatory hormones. The causes are collected in Table 1.

**Table 1:** Endocrine and metabolic causes of Hypoglycemia - Specific pathologies affecting main metabolic and endocrine pathways that can lead to hypoglycemia [5, 9–14, 61, 63]

Hyperinsulinism	Transient Infant of diabetic mother Perinatal asphyxia Rhesus hemolytic disease Intrauterine growth restriction *HNF4A/HNF1A* Congenital *ABCC8/ KCNJ11/ GCK/ GDH/ HADH/ HNF4A/ HNF1A/ UCP2/ SLC16A1/PMM2/HK1/PGM1/FOXA2/CACNA1D/EIF2S3* Others Post-prandial hyperinsulinemic hypoglycemia Insulinoma Munchausen’s by proxy Exercise induced hyperinsulinemic hypoglycemia
Hypoinsulinemic hypoglycemia	Activating *AKT2* mutations
Counter-regulatory hormone deficiency	Growth hormone deficiency Adrenal insufficiency
Fatty acid oxidation disorders	Medium chain acyl-CoA dehydrogenase deficiency Long chain acyl-CoA dehydrogenase deficiency Short chain acyl-CoA dehydrogenase deficiency
Defects in ketone body synthesis/ utilization	HMG CoA synthase deficiency HMG CoA lyase deficiency
Carnitine deficiency (primary and secondary)	Carnitine palmitoyl transferase deficiency (CPT 1 and 2), Carnitine deficiency
Gluconeogenic disorders	Fructose-1, 6-bisphosphatase deficiency, Phosphoenolpyruvate carboxykinase (PEPCK) deficiency Pyruvate carboxylase deficiency
Glycogen storage disorders	Glucose-6-phosphatase deficiency Amylo 1–6 glucosidase deficiency Glycogen synthase deficiency
Defects in glucose transport	GLUT 1/2/3 transporters defects
Other metabolic conditions	Galactosemia, Fructosemia, Tyrosinemia, Glutaric aciduria type 2, Maple syrup urine disease, Propionic academia Adenosine kinase deficiency Mitochondrial respiratory chain disease

Hereditary disorders caused by deficiency of specific enzymes involved in mobilization, interconversion, or utilization of metabolic substrates frequently are associated with hypoglycemia. These enzymatic defects may involve carbohydrate, amino acid, or fat metabolism and are individually rare; almost all are inherited as autosomal recessive traits [8].

## HYPERINSULINEMIC hypoglycemia (HH)

HH is a condition caused by the upregulation of β-cell secretion of insulin producing a hypoglycemic state. Congenital hyperinsulinism (CHH) is the most common cause of transient or permanent hypoglycemia and could potentially be life threatening causing neurological damage. Hence it requires quick and effective treatment and management [8]. This disorder is rare and has an incidence of around 1:40,000 births in the general population [15]. CHH can occur due to genetic mutations and one of the most common causes are defects in the β-cell ATP-sensitive potassium (K_ATP_) channels, known as channelopathies [8]. K_ATP_ channels are comprised of two subunits; the inward rectifying Kir6.2 channels and the sulphonylurea receptor-1, SUR-1, which are encoded for by the *KCNJ11* (potassium voltage-gated channel subfamily J member 11) and *ABCC8 (*ATP-binding cassette transporter sub-family C member 8*)* genes, respectively [16]. Both these subunits are sensitive to the ADP/ATP nucleotide ratio and work together to promote cell depolarization and eventual insulin secretion. Mutations in the *KCNJ11/ABCC8* genes are known to cause defects in biogenesis/trafficking of these subunits to the plasma membrane, thus causing HH.

## Causes of HH

### Transient forms of HH

Transient HH is a poorly defined term that refers to the group of patients in whom HH spontaneously resolves within a few days to approximately a week. However, the cohort includes children requiring medications up to 6 months of life and is usually negative for a known genetic etiology for HH [17]. It is associated with intra-uterine growth retardation, erythroblastosis fetalis, perinatal asphyxia, maternal diabetes mellitus (gestational or insulin dependent) and after the maternal administration of drugs such as sulphonylureas, and intravenous glucose infusions during labor [2]. Abnormal neurodevelopment is evident in one third of children with transient forms of HH associated with perinatal risk factors [5].

### Permanent form of HH

A permanent form of HH, usually congenital (CHH), is where children continue to need medical treatment even after 6 months of age. Various genetic causes have been identified, however nearly 40–50% of children still remain genetically unidentified [17].

#### Molecular basis of CHH

To date, at least 15 genes have been identified to be accompanied with CHH, which include *ABCC8, KCNJ11, GLUD1, GCK, HADH, SLC16A1, UCP2, HNF1A, HNF4A, HK1, PGM1, PMM2, FOXA2, CACNA1D* and *EIF2S3.*

Various modes of inheritance are observed. For some patients specific clinical characteristics, such as the presence of hyperammonemia, can help guide molecular testing; however, for most of the genetic subgroups there is an overlap in phenotype and as such testing of all the known genes is often required.

##### K_ATP_ channel genes (*ABCC8* and *KCNJ11*)

**Fig. 1:**
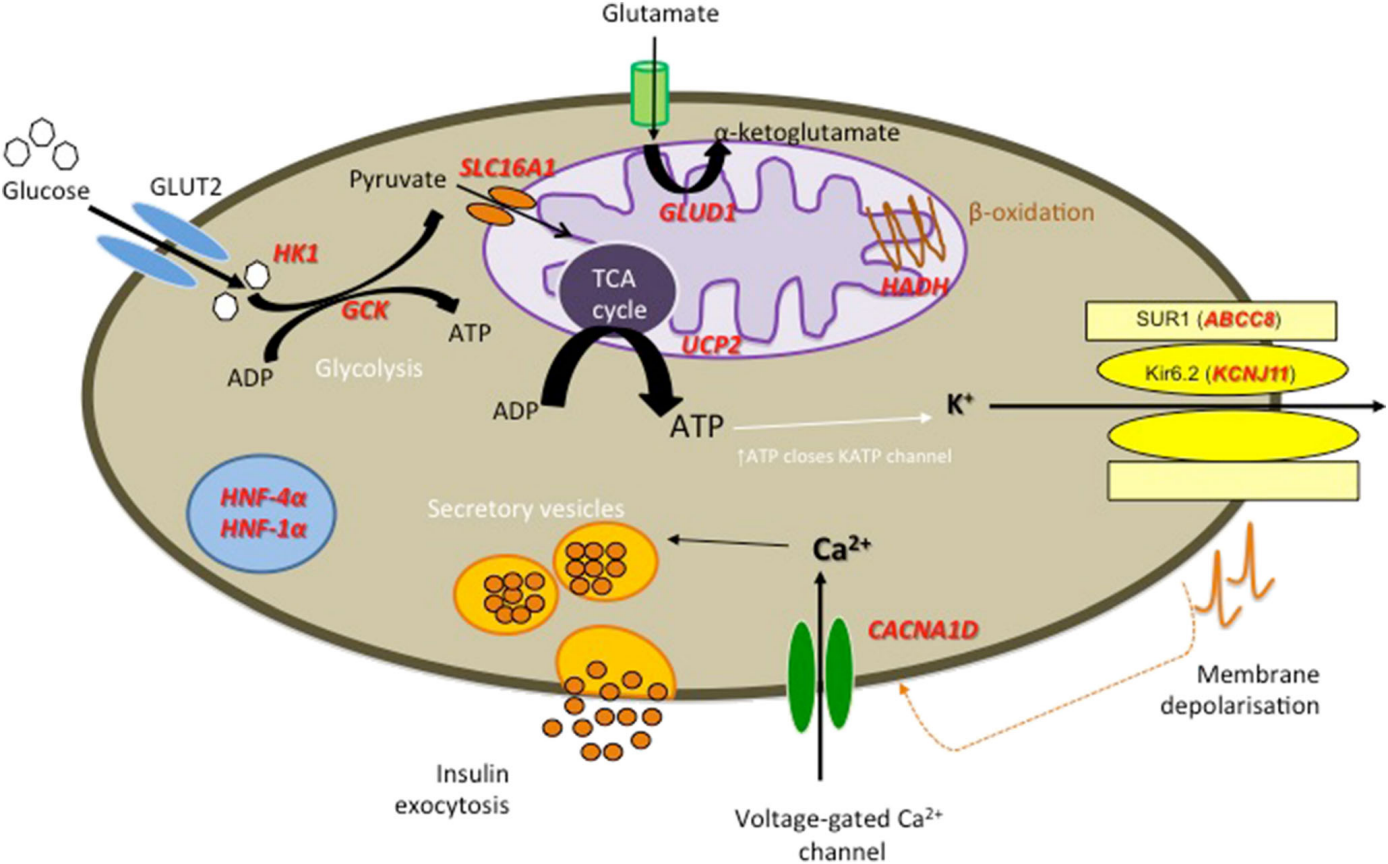
Diagrammatic representation of β-cell function. Genetic defects associated with CHH are included in red. Postprandial glucose is taken into the β-cells via the glucose transporter 2 (GLUT2). Glucose then enters the glycolysis pathway followed by mitochondrial citric acid cycle (TCA) yielding the high-energy molecule, adenosine triphosphate (ATP). ATP molecules travel to and inhibit the potassium-dependent ATP channels (K_ATP_), which prevents influx of potassium resulting in membrane depolarization. This triggers voltage-gated calcium channels to open and influx of calcium (Ca^2+^) occurs. The Ca^2+^ activates the enzyme phospholipase C (PLC) to produce inositol 1, 3, 5 triphosphate (IP_3_) and diacylglycerol (DAG) from phosphatidyl 1, 3 bisphosphate (PIP_2_). The IP3 molecule binds to the protein receptor on the endoplasmic reticulum (ER) to promote a release of Ca^2+^ from the ER. This subsequently increase in cytoplasmic Ca^2+^ promotes exocytosis of the pre-packaged mature insulin and active C-peptide, which are released into circulation. GLUT2: Glucose transporter 2; Glucokinase (GCK) encoded by *GCK* gene; ADP: Adenosine diphosphate; ATP: Adenosine triphosphate; Monocarboxylate transporter (MCT1) encoded by *SLC16A1* gene; Glutamate dehydrogenase (GDH) encoded by *GLUD1* gene; Uncoupling protein 2 (UCP2) encoded by *UCP2* gene; L-3-hydroxyacyl-coenzyme A dehydrogenase (HADH) encoded by *HADH* gene; SUR1 subunit of the K_ATP_ channel encoded by the *ABCC8* gene; Kir6.2 subunit of the K_ATP_ channel encoded by *KCNJ11* gene; Hepatocyte nuclear factor 4α (HNF4α) encoded by *HNF4A* gene; Hepatocyte nuclear factor 1α (HNF1α) encoded by *HNF1A* gene; HK1: Hexokinase 1 encoded by the gene *HK1*; CACNA1D: calcium voltage-gated channel subunit alpha1 D. Mutations in **Forkhead Box Protein A2 (FOXA2), Phosphoglucomutase 1 (PGM1) and Phosphomannomutase 2 (PMM2)** are not included in the cartoon.

The pancreatic K_ATP_ channel is a key component of the insulin secretion pathway. Following glycolysis ATP binds to and closes the K_ATP_ channel causing membrane depolarization, opening of calcium channel and insulin exocytosis (Fig. 1). Loss of function variants in *ABCC8* and *KCNJ11* are the most common cause of HH accounting for 40–50% of cases [17–19](OMIM #601820 and #256450). These variants exert their effects by 1) leading to a loss of K_ATP_ channels at the plasma membrane via effects on gene expression, protein synthesis, protein maturation, or membrane trafficking or 2) by impairing the ability of SUR1 to regulate channel activity by reducing or abolishing channel activation by MgADP and/or MgATP [20, 21]. Recessively inherited variants are the most common and usually result in medically unresponsive HH. Dominant variants in both genes have also been reported [22–24]. The functionally more severe dominant variants cause diazoxide unresponsive HH requiring near-total pancreatectomy whilst milder variants cause diazoxide-responsive HH [25]. In some of the milder cases a bi-phasic phenotype has been reported whereby HH remits in childhood and diabetes is diagnosed in adulthood [26].

##### Glutamate dehydrogenase (*GLUD1*)

Gain of function mutations in *GLUD1* gene, which encodes the mitochondrial enzyme glutamate dehydrogenase (GDH), cause leucine-sensitive HH (OMIM #606762) [27]. Within the pancreatic β-cell, leucine can activate GDH to catalyze the oxidative deamination of glutamate to α-ketoglutarate and ammonia. A-ketoglutarate then enters the tricarboxylic acid cycle (TCA) which generates ATP, ultimately leading to insulin exocytosis. Disease-causing variants in *GLUD1* cluster around allosteric binding sites and act to reduce the sensitivity of GDH to inhibition by GTP and ATP [28]. This loss of regulation leads to an increase in the activity of GDH, a subsequent increase in the amount of α-ketoglutarate entering the TCA cycle and consequently an unregulated insulin secretory response.

Individuals with *GLUD1* variants usually present with a milder form of HH that is often diagnosed outside of the neonatal period and is diazoxide-responsive [27]. In some patients dietary protein restriction may also be required. A consistent feature of this disorder is the presence of plasma ammonium concentrations raised two to three times the upper limit of normal. The presence of persistent hyperammonemia, in most but not all patients with *GLUD1* variants [29], has led to this subtype of hyperinsulinism being referred to as Hyperinsulinism/Hyperammonemia (HI/HA) syndrome (OMIM #606762). An increased risk of epilepsy has also been observed in individuals with disease-causing *GLUD1* variants [29].

In the majority of cases *GLUD1* variants arise *de novo,* with no family history of hyperinsulinism. In keeping with their dominant nature, 50% of future offspring of affected individuals are at risk of inheriting the variant and developing HH.

##### Glucokinase (*GCK*)

Within the pancreatic β-cell glucokinase (*GCK*) plays a key role in linking insulin secretion to a glucose challenge by facilitating the phosphorylation of glucose to glucose-6-phosphate, the first step in glycolysis. Heterozygous gain-of-function variants in *GCK* cause HH by increasing the affinity of GCK for glucose which then acts to lower the threshold for glucose-stimulated insulin secretion [30] (OMIM #602485).

Individuals with gain-of-function *GCK* variants will often have a dominant family history of HH. The absence of a family history should however not preclude testing as *de novo* variants have been reported [31]. Variability in the severity of HH is also observed both in terms of age at presentation, which can range from birth to adulthood, and treatment response [31, 32]. Whilst the majority of individuals are successfully treated with diazoxide, some patients have medically unresponsive HH and require near-total pancreatectomy [32]. Although these differences in phenotype are likely to correlate with the functional severity of the variant, phenotypic variability within families with the same variant has been observed and is likely to be a consequence of genetic background and/or environmental factors [33].

##### Hydroxyacyl-coenzyme a dehydrogenase (*HADH*)

HADH encodes 3-Hydroxyacyl-coenzyme A dehydrogenase, an intramitochondrial enzyme that catalyzes the penultimate reaction in the β-oxidation pathway. Loss-of-function variants in *HADH* result in loss of interaction between HADH and glutamate dehydrogenase [34, 35]. This in turn leads to an increase in glutamate dehydrogenase activity, a subsequent rise in intracellular ATP and upregulated insulin secretion [36].

Patients with *HADH* disease-causing variants present with diazoxide-responsive protein-induced HH [36–38](OMIM #609975). The severity in phenotype ranges from mild late onset hypoglycemia to severe neonatal hypoglycemia. In some patients there are raised plasma concentrations of 3-hydroxybutyrylcarnitine and urinary 3-hydroxyglutaric acid [36]. Loss- of- function variants in *HADH* are recessively inherited and this is the most common genetic subtype of HH in consanguineous individuals [39].

##### Hepatocyte nuclear factors (*HNF4A* and *HNF1A*)

The hepatocyte nuclear transcription factors, HNF1A and HNF4A, play crucial roles in glucose-stimulated insulin secretion as evidenced by the identification of loss-of- function variants in these genes in individuals with maturity-onset diabetes of the young (MODY)(OMIM #125850), an autosomal dominant form of diabetes typically diagnosed before the age of 25 years [40]. *HNF4A* variants were reported to cause a bi-phasic phenotype in individuals presenting with macrosomia and transient HH during the neonatal period and diabetes in later life [41]. The duration of HH varies markedly with some patients treated with intravenous glucose infusion for 1–2 days, yet others require diazoxide treatment for up to 11 years [42–44]. HH with Fanconi syndrome, a renal tubular dysfunction, has been reported in at least 8 patients with *HNF4A* HH, all individuals have the p.R76W variant suggesting that this is a mutation specific phenotype [45–48]. A few cases with *HNF1A* variants and transitory neonatal hypoglycemia have also been reported [45, 49].

Whilst a dominant family history of macrosomia, neonatal hypoglycemia and/or young onset diabetes can help to guide genetic testing for this condition, the absence of affected family members should not preclude analysis of *HNF4A* as *de novo* disease-causing variants have been reported [43].

##### Solute carrier family 16 member 1 (*SLC16A1*)

*SLC16A1* gene encodes the monocarboxylate transporter (MCT1) which transports the insulin secretagogues pyruvate and lactate. Under normal physiological conditions *SLC16A1* is not expressed in the β-cell thus preventing insulin from being secreted in response to lactate and pyruvate. Rare activating dominant variants result in the expression of MCT1 in the β-cell leading to pyruvate-stimulated insulin secretion following exercise [50, 51] (OMIM #610021). For patients with exercise-induced HH, treatment is not usually necessary as hypoglycemic episodes may be prevented by avoiding strenuous exercise [52].

##### Uncoupling protein 2 (UCP2)

*UCP2* gene encodes an inner mitochondrial carrier protein UCP2 which is widely expressed in tissues, including pancreatic islets [53, 54]. UCP2 can inhibit ATP generation by causing proton leak across the inner mitochondrial membrane and negatively regulates glucose-mediated insulin secretion [54]. Inactivating heterozygous mutations in the *UCP2* gene can enhance glucose oxidation and increase intracellular ATP synthesis leading to HH [54]. *UCP2* mutations can present with either transient or prolonged HH [55]. However, in a recent published study, no mutations were detected in the *UCP2* gene among 206 diazoxide responsive patients [56], suggesting that the role of UCP2 in HH needs further investigation.

##### Hexokinase 1 (HK1)

*HK1* encodes the hexokinase HK1, which catalyzes the phosphorylation of glucose to glucose-6-phosphate as substrate for glycolysis. A family with dominant gain-of-function mutation in the *HK1* gene has been reported with “idiopathic hypoglycemia of infancy” [57]. *In vitro* studies evaluating pancreatic β-cells from CHH patients have shown inappropriate expression of HK1. These pancreatic tissues showed functional K_ATP_ channels with inappropriate secretion of insulin at low plasma glucose concentrations (1 mmol/L) [58].

##### Phosphoglucomutase 1 (PGM1)

PGM1 is involved in glycogen metabolism and is responsible for reversible conversion of glucose-6-phosphate to glucose-1-phosphate. *PGM1* gene encodes the enzyme PGM1 and recessive loss-of-function mutations in *PGM1* cause hypoglycemia [59].

Children with *PGM1* mutation presented with postprandial HH and fasting hyperketotic hypoglycemia [59].

##### Phosphomannomutase 2 (PMM2)

Recessively inherited variants in *PMM2*, which encodes a key enzyme in N-glycosylation, have been identified in individuals with HH and polycystic kidney disease. In all individuals a c.-167G > T promotor variant was identified that was either homozygous or *in trans* with a coding variant [60].

The majority of patients with *PMM2* variants present with macrosomia at birth and hypoglycemia in the first year of life. For many, hypoglycemia was the presenting feature and often manifested with seizures. Patients are responsive to treatment with diazoxide [60].

##### Forkhead box protein A2 (FOXA2)

Mutations in *FOXA2* have been reported to cause hypopituitarism, CHH and endoderm-derived organ abnormalities. These children have a unique clinical phenotype of hypopituitarism, CHH, dysmorphic features, and liver, pancreas, heart and gastrointestinal abnormalities [61, 62].

##### **C**alcium voltage-gated channel subunit alpha1 D (CACNA1D)

*CACNA1D* gene encodes an L-type voltage-gated calcium channel which is expressed in pancreatic β-cells and regulates insulin secretion. Mutations in *CACNA1D* have been reported to cause HH, heart defects and severe hypotonia [63].

##### Eukaryotic translation initiation factor 2 subunit 3 (EIF2S3)

Three cases published with variant in *EIF2S3* present an unusual dysregulation of glucose fluctuating between diazoxide-responsive HH and postprandial hyperglycemia, along with learning difficulties and hypopituitarism [64].

### Other forms

#### Postprandial forms of HH

In postprandial hyperinsulinemic hypoglycemia (PPHH), hypoglycemia is induced hours after meal intake due to inappropriate/exaggerated insulin secretion in response to the meal. The most common cause is “dumping” syndrome in infants/children who have undergone Nissen fundoplication/gastric bypass [65, 66]. Children with PPHH after Nissen fundoplication have an abnormally exaggerated secretion of the insulin secretagogue glucagon-like peptide 1 (GLP-1) which may contribute to the exaggerated insulin surge and resultant hypoglycemia [67].

PPHH is also reported in the insulin autoimmune syndrome leading to development of insulin binding autoantibodies in children who were not previously exposed to exogenous insulin [68].

#### Other causes of HH

Insulinoma is a rare cause of hyperinsulinism and must be considered in older children or adolescents presenting with HH [69]. A detailed family history of tumors especially like insulinoma, is relevant as it can be part of multiple endocrine neoplasia syndrome type 1 (MEN1).

*Munchausen by proxy* leading to exogenous administration of insulin or anti-diabetic drugs such as sulphonylureas can present as factitious HH. This has led to misdiagnosis and consequent pancreatectomy [70].

#### Syndromes associated with HH

A large number of syndromes are associated with HH, with Beckwith-Wiedemann syndrome (BWS) being one of the commonest [71]. Table 2 lists the syndromic associations with HH, although the exact mechanism for HH is still not well understood in all the conditions. Most syndromic forms are diazoxide-reponsive and HH resolves overtime.

**Table 2:** Syndromic forms of HH - Various developmental syndromes have been described with the gene/s linked to the condition and the common clinical features [203]

SYNDROME NAME	GENETIC ETIOLOGY *gene* (location)	CLINICAL CHARACTERISTICS
Pre- and postnatal overgrowth (Macrosomia)		
Beckwith-Wiedemann	(11p15)	Macroglossia, abdominal wall defects, ear lobe pits/ creases, hemihypertrophy, tumor risk
Sotos	*NSD1* (5q35)	Macrocephaly, frontal bossing, pointed chin, developmental delay, tumor risk
Simpson-Golabi-Behmel	*GPC3* (Xq26), *GPC4* (Xp22)	Coarse facial features, broad feet, polydactyly, cryptorchidism, hepatomegaly, tumor risk
Perlman	*DIS3L2* (2q37)	Inverted V-shaped upper lip, prominent forehead, developmental delay, hypotonia, tumor risk
Postnatal growth failure (short stature)		
Kabuki	*KMT2D* (12q13), *KDM6A* (Xp11.3)	Arched eyebrows, long eyelashes, developmental delay, fetal finger pads, scoliosis, heart defects, hypotonia
Costello	*HRAS (*11p15*)*	Deep palmar/plantar creases, developmental delay coarse facial features, heart abnormalities, papillomas, tumor risk
Chromosomal abnormality		
Mosaic Turner	(Loss of X in some cells)	Milder Turner syndrome phenotype (short stature, coarctation of aorta, gonadal dysgenesis)
Patau	Trisomy 13	Developmental delay, microphthalmia, heart & neural defects
Congenital disorders of glycosylation		
Types 1a, 1b, and 1d	*PMM2* (16p13.2), *MPI* (15q24.1), *ALG3* (3q27.1)	Developmental delay, hypotonia, growth failure
Contiguous gene deletion affecting the *ABCC8* gene		
Usher	11 genes	Hearing loss, visual impairment
Abnormalities in calcium homoeostasis		
Timothy	*CACNA1C* (12p13.33)	Long QT syndrome, syndactyly, developmental delay, immune deficiency
Insulin receptor mutation:		
Insulin resistance syndrome (leprechaunism)	*INS* (19p13)	Hypo- and hyperglycemia, pre- and postnatal growth restriction, elfin-like features, hirsutism
Other Syndromes:		
Congenital central hypoventilation syndrome	*PHOX2B* (4p13)	Central hypoventilation, “box-shaped” face, neurocristopathies (Hirschsprung disease, tumor risk)

## Pathophysiology of HH - histological subtypes

**Fig. 2:**
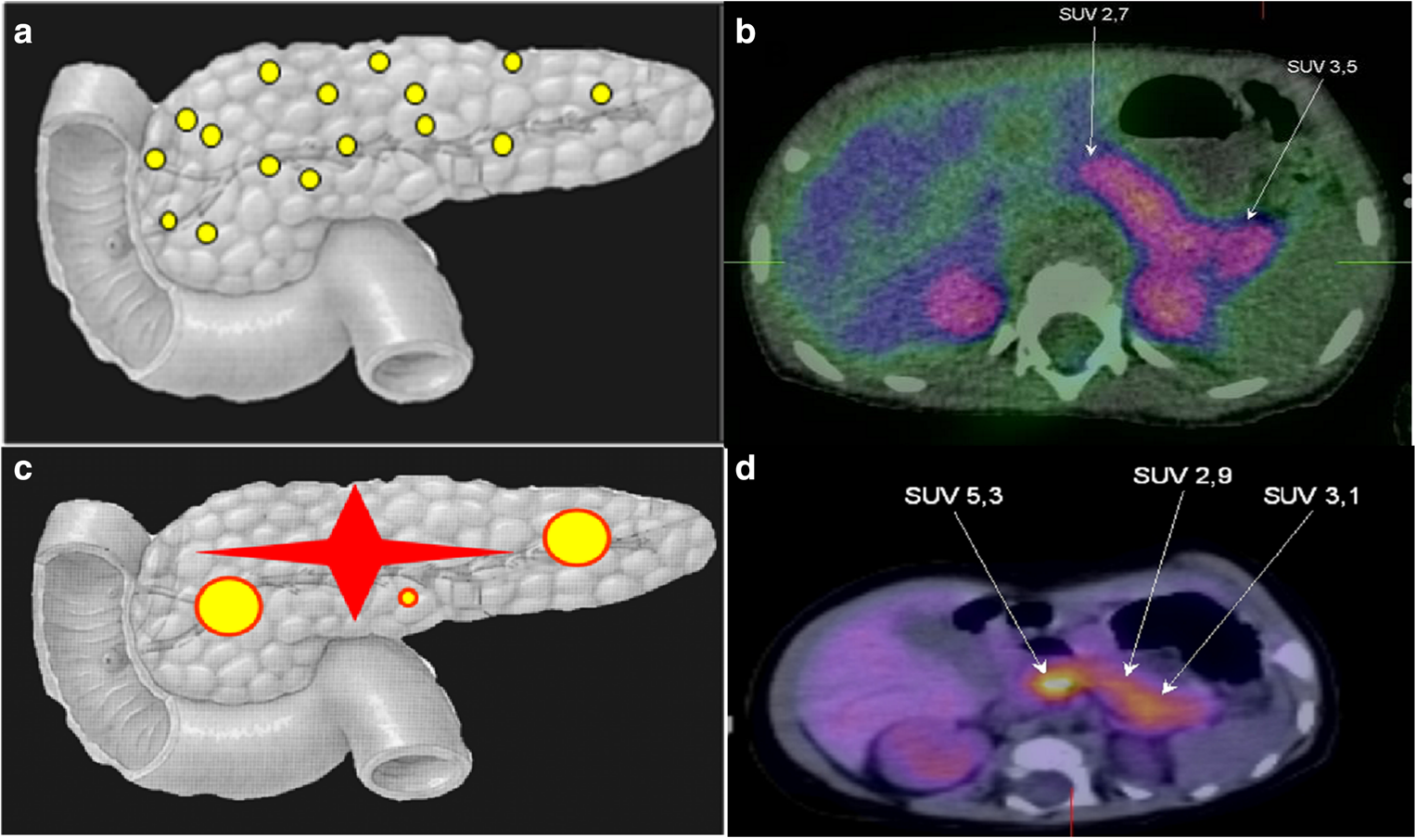
Diffuse and focal form of HH with 18F-DOPA-PET-CT images. A – Diagrammatic representation of diffuse form of CHH and B – 18F-DOPA-PET image of diffuse form of CHH. C – Diagrammatic representation of focal form of CHH (showing different types of focal lesions) and D – 18F-DOPA-PET-CT image of focal lesion in the head of pancreas. SUV – Standardized uptake value.

There are three histological forms of CHH (Fig. 2); Focal form (F-CHH), Diffuse form (D-CHH) and atypical. The clinical presentation appears to be similar, although their molecular mechanisms are quite different. In most cases D-CHH is inherited in an autosomal recessive manner whereas the F-CHH is sporadic [22, 72].

### Focal form of CHH (F-CHH)

Focal lesions occur when the abnormal pancreatic β-cells are localized to a single specific location in the pancreas. They are the result of two unique events, first the inheritance of a paternally inherited *ABCC8* or *KCNJ11* mutation at 11p15.1, and secondly the loss of the corresponding maternal allele within the focal lesion. This causes an imbalance in the expression of imprinted genes such as the maternally expressed tumor suppressor gene *H19* and *CDKN1C*, and the paternally expressed growth factor *IGF-2*, at 11p15.5 [73] leading to β-cell hyperplasia. Within the focal islet cell lesion there is adenomatous hyperplasia and abnormally large nuclei in the affected cells.

A duplication of the paternal allele located on chromosome 11 has also been found in some patients with F-CHH [74]. Outside the lesion, normal endocrine tissue with small nuclei exists. These cells appear to have less cytoplasm and diminished proinsulin production [75–78].

Patients who have a heterozygous paternally inherited mutation in *ABCC8* or *KCNJ11* could have F-CHH, which accounts for 30–40% of all CHH cases [79]. F-CHH is confirmed by a fluorine-18 dihydroxyphenylalanine-positron emission tomography (18F-DOPA-PET) scan, which can show the presence of a focal lesion and determine its location with diagnostic accuracy [80].

### Diffuse form of CHH (D-CHH)

The D-CHH form occurs when all the islet cells in the pancreas are abnormal [77, 81]. Patients with a homozygous recessive or a compound heterozygote mutation in *ABCC8* or *KCNJ11* present with D-CHH. These patients are usually medically unresponsive, and this histological form accounts for 60–70% of all CHH cases. Most islets throughout the entire pancreas are affected with the presence of large hyperchromatic nuclei [75–78, 81].

### Atypical forms of HH

Histologically atypical forms of CHH are categorized when the pancreatic morphology does not fit into the F-CHH or D-CHH types and are a mosaic pattern of the two [73, 82]. The islets can either be enlarged or shrunken. Some cases have been cured with a lesionectomy whilst others also require medical management. However, to date only one patient has been described with a *ABCC8* nonsense mutation (Q54X) causing this histological form of CHH [82]. Studies have shown that the heterogeneous expression of β-cells HK1 in the atypical CHH may be causing the abnormal insulin release [83, 84].

## Clinical presentation of HH

HH usually manifests in infancy or early childhood, although some patients can present during adolescence or adulthood. Signs and symptoms of hypoglycemia are non-specific during the neonatal period (poor feeding, jitteriness, irritability), challenging its diagnosis. At later ages, symptoms can be easier to recognize and can be classified as adrenergic (hunger, pallor, tachycardia, tremor, diaphoresis) and neuroglycopenic (tiredness, blurred vision, confusion, coma and even death).

Risk factors for HH need to be looked for in the perinatal history. These include maternal intrapartum administration of hypoglycemic agents, stressful delivery, large/low birth weight, and neonatal polycythemia or jaundice, among others. Examination of the baby will search for macrosomia/leanness, careful phenotypic characterization to identify syndromes, features of cardiomyopathy (due to glycogen storage in HH), hepatomegaly present in metabolic conditions (also present in HH), and midline abnormalities (including genitalia) to exclude hypothalamic-pituitary deficits. Data in the family history could suggest a genetic inheritance, therefore anamnesis must enquire for consanguinity, cases of diabetes mellitus, hypoglycemia, seizures and unexplained deaths [85].

## Diagnostic investigations of HH

Any child requiring intravenous glucose load greater than 8 mg/kg/min (normal requirement is 4-6 mg/kg/min) to avoid hypoglycemia, can essentially be labeled as HH [86]. The timing of hypoglycemic events with regard to meals will give insight into the intrinsic mechanism. HH typically manifests during brief periods of fasting, however certain types can be manifest soon after meal ingestion (protein induced HH) or hours after meal ingestion (postprandial HH). Should there be a clear link between physical activity and occurrence of hypoglycemia, then exercise induced HH should be excluded.

### Biochemistry

Biochemical interpretation will only be possible if the critical sample is taken adequately at the time of hypoglycemia (≤3.0 mmol/l), ideally in a controlled fasting setting. HH will demonstrate detectable C-peptide or insulin in the face of hypoglycemia, with simultaneously low/undetectable alternative substrates (ketones and fatty acids) [86, 87]. Of note, the concentration of insulin does not parallel the degree of severity of the condition [88, 89]. Table 3 defines the diagnostic criteria for HH. If a particular mechanism is suspected to trigger HH (exercise, protein, carbohydrate etc) specific stimulation tests will be carried out:

**Table 3:** Diagnostic criteria for HH - The cut-off values for each analyte to aid in the diagnosis of HH. IGFBP-1: Insulin growth factor binding protein-1. HI/HA: Hyperinsulinemic hypoglycemia hyperammonemia syndrome. HADH: short chain L-3-hydroxyacil-CoA dehydrogenase. Im: intramuscular. Iv: intravenous. Sc: subcutaneous *[90, 200, 203]^¥^

Serum analyte	Result in patients with HH
Blood glucose < 3.0 mmol/l (54 mg/dl) and:	
Insulin	Detectable
C-peptide	Detectable (≥0.5 ng/mL^¥^)
Free fatty acids	Low or suppressed (<1.5 mmol/l* or < 1.7 mmol/l^¥^)
Ketone bodies	Low or suppressed (3-β-hydroxybutyrate <2 mmol/l* or < 1.8 mmol/l^¥^)
IGFBP-1	Low (≤110 ng/mL^¥^) as insulin negatively regulates IGFBP-1 expression
Ammonia	Normal. Can be raised in HI/HA syndrome
Hydroxybutyrylcarnitine	Normal. Raised in HH due to *HADH* mutation
Cortisol, Growth hormone	Raised. Generally Cortisol >20 μg/dL [500 nmol/L]; growth hormone >7 ng/mL - younger children might have poor counter-regulatory response
Amino acids and urine organic acids	Normal. Leucine, isoleucine and valine may be suppressed in HH
Proinsulin	>20 pmol/l
Additional information when diagnosis of HH uncertain:	
Glucose infusion rate	>8 mg/kg/min to achieve euglycemia
Im or iv glucagon administration or sc octreotide administration	>1.5 mmol/L or 27 mg/dl (Positive glycemic response)

#### Protein load test

In this test leucine or a combination of aminoacids is administered enterally. Evidence of raised insulin concentrations at the time of hypoglycemia will confirm the clinical suspicion of protein sensitive HH [29].

#### Oral glucose tolerance test / mixed meal test

Enteral administration of a mixed meal or oral glucose load followed by hypoglycemia with a detectable insulin within 2–5 h, indicates incretin mediation leading to postprandial HH. In adults the prefered test is the standardized hyperglucidic breakfast [91], which has not yet been standardized in children.

#### Exercise test and pyruvate load

Biochemical evidence of HH during these tests indicate a lactate/pyruvate mechanism triggering exercise induced HH [92].

#### Fructose load test

Individuals with ketotic hypoglycemia after fructose ingestion should undertake a fructose load test to identify hereditary fructose intolerance and fructose-1,6-diphosphatase deficiency, and this can be followed by genetic testing.

#### The role of real-time continuous glucose monitoring (CGM)

CGM measures interstitial glucose concentrations indicating the direction, magnitude, frequency, duration and causes of fluctuations in these. There is now a study on its accuracy in children with HH [93], as this can significantly improve the quality of life of these patients.

### Imaging

#### Recent advances in diagnostic imaging of HH

As is not possible to differentiate between F-CHH and D-CHH based on clinical presentation and biochemical features, pre-operative differentiation of both subtypes is critically important. Conventional radiological imaging such as Magnetic Resonance Imaging (MRI) and Computerized Tomography (CT) scans fail to localize the focal lesions. Genetic analysis for mutations in *ABCC8/KCNJ11*, with combination of recently described Fluorine 18 L-3, 4- dihydroxyphenyalanine positron emission tomography (18F-DOPA-PET-CT) scanning, allows differentiation between F-CHH and D-CHH with a high sensitivity (88%) and specificity (94%) [94–98], with an accuracy of 100% [99–102]. The principle of 18F-DOPA-PET analysis is based on the selective uptake of L-DOPA by β-cells and its conversion into dopamine by DOPA decarboxylase enzyme, which is expressed in the pancreatic β-cells. Thus, imaging with 18F-DOPA-PET-CT should be performed in all patients who are thought to have F-CHH. Some patients have been described with atypical histological forms, which are either atypical focal, atypical diffuse or ectopic β-cell hyperplasia and do not show classical features of F-CHH or D-CHH [103]. Demonstration of increased activity of DOPA decarboxylase by 18F-DOPA-PET in combination with an enhanced CT imaging can successfully differentiate diffuse and focal β-cell hyperplasia. Therefore, this technique has radically changed the surgical approach to patients with medically unresponsive HH [102, 104].

A dosimetry study of 18F-DOPA derived from the PET-CT images in ten infants (median age 4.84 weeks) with HH suggested that a modest radiation dose (0.30 ± 0.04 mSv/MBq) was adequate [105]. However, there have been recent reports of its inaccuracy in precisely detecting focal lesions [106, 107]. Glucagon-like-peptide-1 (GLP-1) receptor analogs are the latest agents being used in the detection of insulinomas in adults [108, 109]. Similarly, this isotope is being investigated in children to detect focal lesions.

#### Imaging for insulinoma

Most insulinomas are benign (islet-cell tumors) and usually very small (<2 cm) making it difficult to localize with the current imaging techniques. Various non-invasive techniques have been used for the detection of insulinomas e.g., transabdominal ultrasonography, spiral CT, MRI, ^111^In-pentetreotide imaging, and ^18^F-l-dihydroxyphenylalanine PET. In difficult cases, invasive procedures like endoscopic ultrasonography or selective arterial calcium stimulation test with hepatic venous sampling have also been used [110].

A systematic review and meta-analysis of (68)Ga-DOTATATE compared with octreotide and conventional imaging documented a high sensitivity (90.9%) and specificity (90.6%) of (68)Ga-DOTATATE in detecting neuroendocrine tumors [111]. Various Gallium based isotopes have also been used to detect insulinoma in adults [108, 112].

## Management of HH

Maintaining normoglycemia (blood glucose >3.5 mmol/l) is paramount to avoid hypoglycemic brain injury in view of the hypoketotic nature of HH [113]. This may be achieved by increasing glucose administration (feeds or intravenous (iv) fluids), stimulating endogenous glucose release (glucagon administration) or suppressing insulin release from the β-cell (diazoxide, octreotide, nifedipine). Oral feeds with additional glucose polymer in combination with iv fluids can also be used to maintain normoglycemia. The traditional and new drugs available for the management of HH are summarized in Table 4.

**Table 4:** Standard and novel drugs used in the management of HH - Medications used for the treatment of HH, along with their dose, mechanism of action and side effects [114–116, 134, 140, 201]

Medication *(route of administration)*		Total daily dose	Action mechanism	Side effects
**Standard drugs**	**Diazoxide** *(enteral)*	5-20 mg/kg/day (divided in 3 doses)	Binds to the SUR1 subunit of intact KATP channels, opening the channel and inhibiting insulin release	Common: Fluid and sodium retention, hypertrichosis, anorexia. Rare: Cardiac failure, pulmonary hypertension**, blood dyscrasia, hyperuricemia, paradoxical hypoglycemia
	**Chlorothiazide** *(enteral)*	7-10 mg/kg/day (divided in 2 doses)	Synergy with diazoxide over KATP channels inhibiting insulin secretion. Prevents fluid overload	Hyponatremia, hypokalemia
	**Glucagon** *(sc/im bolus; sc/iv infusion)*	Bolus: 0.02 mg/kg/dose Infusion: 2.5–10 mcg/kg/h	Stimulates glycogenolysis, gluconeogenesis, ketogenesis, lipolysis	Skin rash, vomiting. Paradoxical rebound hipoglycemia if dose >20mcg/kg/h (high dose stimulates insulin release)
	**Octreotide** *(sc)*	5–40 mcg/kg/day (divided in 3–4 doses or continuous infusion)	Activation of SSTR-2 and SSTR-5. Stabilisation of K_ATP_ channel, reduces calcium entry in β-cell, inhibition of insulin secretion. Inhibitition of INS promoter.	Acute: Abdominal discomfort, vomiting, diarrhea, anorexia, hepatitis, transaminasemia, long QT syndrome, necrotizing enterocolitis, tachyphylaxis. Long-term: Cholelithiasis, intestinal hypomobility, suppression of GH and TSH
	**Nifedipine** *(enteral)*	0.25–2.5 mg/kg/day (divided in 2–3 doses)	Blockage of β-cell calcium channel activity, leading to inhibition of insulin exocytosis	Hypotension
	**Acarbose** *(enteral)*	6.25–300 mg/day (divided in 3 doses – before main meals)	Inhibits intestinal α-glucosidase (cleaves polysaccharides to monosaccharides)	Intestinal discomfort, diarrhoea, flatulence, raised transaminases
**Novel drugs**	**Lanreotide and long-acting octreotide** *(deep sc or im)*	30–60 (max 120*) mg/dose (every 4 weeks)	Like octreotide. High affinity for SSTR 2 & 5, and reduced affinity for SSTR 1, 3 & 4	Same as octreotide. Pain at injection site. No long-term data available yet.
	**mTOR inhibitors** *(enteral)*	Starting dose: 1 mg/m^2^/day (divided in 2 doses). Adjust dose aiming for blood concentrations 5-15 ng/ml	Inhibits mTOR complex 1. Inhibits β-cell proliferation and insulin secretion. Posible induction of peripheral insulin resistance	Immune suppression, hyperlipidemia, hypertransaminasemia, mucositis, thrombocytosis

Sc: subcutaneous. Im: intramuscular. Iv: intravenous. SSTR: Somatostatin receptor. INS: insulin gene. SUR1 = sulfonylurea receptor 1. KATP = ATP-sensitive potassium channel.

**[115, 116]

*90-120 mg every 4 weeks [134]*.* The starting dose of Lanreotide autogel 30 mg has been found to be effective [132, 133]

## Current medical management

### Acute management of hypoglycemia

If the oral route is unavailable or glycemia is not improving despite oral glucose (glucose gel/glucose containing drinks or tablets), then an iv dextrose bolus (2 ml/kg 10%Glucose) needs to be administered followed by continuous iv glucose infusion (>8 mg/kg/min). If there is persistence of hypoglycemia, hypoglycemic seizure or inadequate iv access, then an intramuscular or subcutaneous bolus (1 mg) or infusion of glucagon can be life saving as it causes immediate release of glycogen stores from the liver leading to a temporary improvement in blood glucose concentrations. [117]*.*

As high iv glucose concentration is usually needed for weeks, insertion of a central venous access supports in managing glycemia safely.

### Long-term pharmacological agents

Medical therapies can be tried to wean the infant off iv support and achieve a near-normal feeding pattern. Surgical therapy is considered when trial of medical therapies is unsuccessful or in case of established F-CHH.

HH drug responsiveness is defined by: 1) normal feeding frequency and volume, 2) fasting capability adequate for age whilst maintaining euglycemia, 3) low/suppressed serum insulin level at the end of the fast, and 4) appropriate generation of ketone bodies and fatty acids by the end of the fast [117].

#### Diazoxide

The first drug of choice is diazoxide [86], which requires a functional K_ATP_ channel to bind onto and is hence ineffective in D-CHH due to an inactivating K_ATP_ mutation and in most cases of F-CHH. Most other forms of HH and protracted HH secondary to risk factors such as IUGR and perinatal asphyxia respond to treatment with diazoxide. Due to its fluid retaining properties, diazoxide therapy must be used with caution in infants with HH who are often receiving large volumes of iv/oral fluids to maintain normoglycemia. Fluid restriction prior to starting diazoxide is commonly practiced, along with concomitant use of a thiazide diuretic such as **chlorothiazide**, which also has a synergistic action over the K_ATP_ channels.

#### Medical management of diazoxide unresponsive D-CHH

##### Nifedipine

Authors suggest triyng it before heading to pancreatectomy [118] as there have been isolated case reports on successful treatment of HH with nifedipine [119–121] but experience of large HH centers has generally been disappointing [117, 122, 123]. It is sometimes considered as an add-on drug in partial diazoxide/octreotide resistance, and/or following partial pancreatectomy [122, 124] but rarely used as monotherapy.

##### Octreotide

Octreotide is a second-line treatment for diazoxide-insensitive patients [125, 126]. It may be used in combination with diazoxide and glucagon in cases with partial diazoxide response, or often combined with frequent feeding which may require a gastrostomy to enable high calorie bolus feeding during the day and overnight continuous feeds. As octreotide binds to the somatostatin receptors SSTR-2 and SSTR-5, prolonged use may develop into drug desensitizing caused by internalisation of the receptors [122].

##### Glucagon

Iv or subcutaneous (sc) glucagon [127] may be helpful during the initial stabilization period and before surgery. However, this treatment has not been of long-term benefit. It can also be administered (alone or in combination with Octreotide) to stabilize blood glucose concentrations in the acute management and prevent near-total pancreatectomy in infants with HH [128]. Continuous sc glucagon infusion can frequently complicate by catheter obstructions occurring daily or 2–3 times per week [128].

##### Acarbose

For postprandial HH the first approach is diet modification. This includes: frequent feeds of long-acting carbohydrates, abundant protein and supplements of fibre and fat emulsions [129]. If hypoglycemia persists, acarbose is the preferred medical option as it slows the absorption of glucose into the blood stream, thus avoiding a glycemic peak followed by insulin release [130].

## Novel medical therapies

### Long-acting somatostatin analogues

Lanreotide and Long-acting release octreotide (LAR-Octreotide) are two formulations used in few numbers of patients with HH [131–136] reported not only to be useful in the management of HH in children, but potentially displaying a more stable glycemic control than octreotide [134]. Lanreotide has also been proven useful in managing inoperable F-CHH [137].

### mTOR inhibitors

The intracellular mTOR pathway is involved in β-cell growth and altered insulin secretion in patients with insulinoma [138]. Therefore, mTOR inhibitors such as sirolimus (formerly known as rapamycin) and everolimus have been used to treat this tumor. Although the exact mechanism of sirolimus in HH still needs to be elucidated, it has been hypothesized that the mTOR complex 1 may be overactivated in D-CHH [139]. The first study reported the use of sirolimus in 4 children with severe D-CHH who achieved glycemic control avoiding pancreatectomy and with no major side effects [140]. This has been followed by various case reports of HH in children of various ages who have benefitted from sirolimus [141–145]. However, there have been reports about its severe side effects as well as poor response in others [146–149]. Our center has tried sirolimus on 22 patients with various forms of HH, out of which 21 showed glycemic response, however 19 patients developed side effects, recurrent and frequent infections being the most common infections [150]. There is no clear correlation between genetic etiology and response to sirolimus.

## Potential novel therapies – The future

### GLP-1 antagonists

GLP-1 acts on the β-cell promoting its proliferation and stimulating insulin release [151]. Mouse models with K_ATP_ channel defects leading to HH, significantly improved their glucose concentrations when treated with GLP-1 receptor antagonist (exendin-9-39) [152]. Infusion of exendin-9-39 has been tried in 9 adult patients with HH due to K_ATP_ mutations [153], where all patients demonstrated increased fasting mean glucose and glucose area under the curve, raising expectation for its near-future use in children with this condition.

### Pharmacological trafficking chaperones

Some mutations in the *ABCC8* gene prevent trafficking of parts of the SUR1 channel from the endoplasmic reticulum to the surface of the cell. Carbamazepine [154] and sulfonylureas [155] – glibenclamide and tolbutamide - are K_ATP_ channel inhibitors and chaperones that have proven to amend channel trafficking defects in many *ABCC8* mutations by allowing intersubunit interactions between SUR1 and Kir6.2 [156]. This has been now confirmed in mouse functional studies as well [157] reinforcing its potential for clinical use.

### Glucagon – New formulations and delivery

Subcutaneous glucagon infusion via pump has been used as long-term treatment for HH patients at home [158]. Practical issues with its administration remain unsolved as it can cause mechanical obstruction due to its formulation. However, a stable form of glucagon formulation could be a potential useful tool that is being explored [159, 160].

## Surgical management

### Surgery for F-CHH

Removal of the affected part of the pancreas achieving complete cure is the surgical aim for the F-CHH. A multidisciplinary team (endocrinology, radiology, and histopathology) should be involved in managing patients with F-CHH and D-CHH [161, 162]. In most cases, pre-operative 18F-DOPA PET-CT helps exact localization of the focal lesion and aids the surgeon. Intra-operative biopsies are important to ensure complete excision with histological confirmation of clear margins. Laparoscopic lesionectomy is the preferred surgical approach when the focal lesion is easily accessible (i.e. body or tail of pancreas) offering the benefit of shorter post-operative care [163]. Focal lesions that are difficult to access such as in the head of the pancreas usually require open laparotomy for resection of most of the pancreatic head and Roux-en-Y pancreaticojejunostomy [164].

### Surgery for D-CHH

Near-total pancreatectomy (95–98% of the pancreas), the only option for medically unresponsive D-CHH has largely been reported to have unsatisfactory outcomes [165, 166]. In a large study, nearly 59% of patients that underwent near total pancreatectomy continued to experience hypoglycemia, though it was usually easier to manage with dietetic/medical therapy. Post-operatively, hyperglycemia was common as well with increasing incidence as age progressed and a 100% incidence by 13 years of age [166]. Complex glucose derangements with persisting fasting hypoglycemia and post-prandial hyperglycemia were also reported in 35% of patients. Exocrine pancreatic insufficiency is also common following near-total pancreatectomy for D-CHH [166]. Laparoscopic surgery is now the preferred option as opposed to the traditional open approach [161, 167].

### Surgery for insulinoma

Surgical removal of the tumor is the mainstay therapy for childhood and adult insulinoma, and its overall cure rate is up to 98% [168, 169]. Prognosis depends on the tumor stage at the time of presentation and success rate of complete resection. The mode of surgery depends on the tumor size, localization, and metastatic characteristics [69, 170]. For small benign tumors with no metastasis that are located at least 2–3 mm from the main pancreatic duct, a limited enucleation should be performed [171]. A tumor that is invading the pancreatic duct or great vessels with risk of malignancy and lymph node invasion, and that is compressing the distal pancreatic duct, might require a more extensive surgical resection. The surgical resection procedure depends on the site of the insulinoma and includes mid-body pancreatectomy, distal pancreatectomy, or pylorus-preserving Whipple procedure [171, 172].

## HH monitoring and follow up

Children with HH need regular monitoring of their blood glucose at home. The follow-up of these children requires involvement of a multidisciplinary team including pediatric endocrinologist, dietitian, nurse specialist, clinical psychologist, developmental pediatrician and pediatric surgeon. Severity and treatment requirement should be assessed periodically in all children on medical therapy and the dose of medications should be optimized based on their glucose monitoring and fast tolerance test. It has been reported that for most patients with K_ATP_ channel mutations who are managed by medical treatment only, severity is reduced over time [173]*.* Children need to be monitored for the side effects of the HH medications [135, 146, 147, 174, 175]. Also, the majority of children with severe HH are on high carbohydrate feeds administered via gastrostomy by bolus and/or continuous feeds, hence the importance in receiving support from the community i.e. nursery, school, home etc. in these group of children. Post-surgical patients require periodical monitoring including stool elastase, capillary glucose measurements and oral glucose tolerance test looking for complications (hypoglycemia, diabetes and exocrine pancreatic insufficiency) [165, 166]. Various studies evaluating the long-term outcome of patients with HH have reported a high frequency of neurodevelopment delay and various neurological disorders, including epilepsy and microcephaly [5, 176, 177]. This group of children may need a special education plan devised with the help of the educational psychologist and developmental pediatrician.

## Transition into adult service

Young adults with HH have complex management requirements that are best supported using a multidisciplinary approach [178]. The move from pediatric to adult services may be a challenging time for adolescents transitioning to self-management [179].

A transition service aims to bridge this changeover in care, and support the adolescent [180] in terms of their health needs, but also psychosocial development, including ability to achieve independence and establish adult relationships [181, 182]. This specific age group are recognized to have barriers that prevent optimal self-management, including [183];

- heightened concerns about peer relationships and social interactions,
- frustration and fatigue from the management of a chronic illness,
- incomplete knowledge and understanding of chronic disease management,
- inclination towards risk-taking and
- difficulties in the transition to self-management.

People with chronic conditions experience differences in the clinical management approach of pediatric and adult care settings during the transition process [182]. Pediatric teams may overlook the growing independence of the individual, however conversely, the encouragement to take responsibility from adult care providers may lead to physical, psychological and social development being neglected [182, 184, 185].

Consequently, young adults often feel misplaced in adult services and this has been shown to lead to lower rates of follow-up appointments, attendance and medication compliance [181, 186].

Discharge to a non-specialist adult team carries with it some challenges, including lack of awareness about this rare condition and its complex on-going health-needs. In addition, non-specialist teams may not be best placed to undertake discussions around pre-conception counselling in those with a confirmed genetic etiology.

### Aims of the transition service

Recognizing the core attributes of this age group, a transition service for patients with HH may be established, with the following aims and in line with established NICE guidance [178].

1. **Supporting education:** Establish and support understanding of HH. Where genetic causes are identified, patients should be aware of this and have an understanding of its inheritance.
2. **Empowering self-management:** To support handover of care to the affected individual from the carers [187], by encouraging shared decision-making and empowering self-management, with an ultimate aim of self-management [188].
3. **Addressing parental and care-giver concern** [189]: Establishing an understanding between the young person and their carer of how aspects of self-care might be jointly undertaken and transferred is an important component of managing distress, expectations and, ultimately, transition [190].
4. **Addressing on-going medical issues:** Table 5 summarizes some of the medical issues that may be addressed in a transition service. The risk and progression to diabetes is variable and appropriate treatments for diabetes may depend on both the mutation causing HH and the extent of β-cell failure.
5. **Dealing with impaired awareness of hypoglycemia** [191]: If found in severe cases of HH it can be formally assessed using the validated Clarke [192] or Gold score [193]. The evidence base for managing impaired awareness of hypoglycemia is limited in the context of HH [194] and so, care is needed in managing these individuals. CGM could be of use in some cases [191].
6. **Providing advice around driving:** Supporting individuals at high risk of hypoglycemia or impaired awareness of hypoglycemia to apply for a driving license may be explored, and fully explaining driving regulations in relation to hypoglycemia is important for those starting to learn to drive [195].
7. **Providing support to higher educational institutions and those in employment** [196, 197]: Flexibility may be required to allow time for testing, or additional breaks for calorie consumption.
8. **Pre-conception genetic counseling, where appropriate:** In those with confirmed dominant mutations, ensuring adequate counseling around offspring, and accessing genetic counselors when appropriate, is important. Insulin modulates Sertoli cell function and young hyperinsulinemic patients have been found to have lower anti-Müllerian hormone and inhibin B secretion [198]. This could therefore influence testicular function and have a future impact on fertility, which still needs to be studied.
9. **Continued dietetic advice:** On-going advice about dietary management of hypoglycemia, especially where there is protein-induced hypoglycemia, and replacement of calories with other macronutrients may be provided.
10. **Alcohol and recreational drug advice** [199]: alcohol consumption can potentiate the risk of hypoglycemia and also impair awareness of hypoglycemia. Recreational drugs may also mask the adrenergic symptoms of hypoglycemia.

**Table 5:** Summary of medical issues encountered in adolescent and young adult patients with congenital hyperinsulinism and the intervention required.

Medical Issue	Support / Intervention needed
Confirmed mutation causing HH	
Symptom control	• Exploring precipitants • Dietary interventions and advice • Need for medical treatment e.g. diazoxide, calcium channel blockers, somatostatin analogues. • Accessing appropriate technology e.g. real-time CGM, where appropriate
Risk of diabetes	• Aware of diabetes symptoms • Annual glucose checks • Understands the risk of diabetes
Managing diabetes in non-pancreatectomized individuals	• Establishing type of diabetes • Impact of underlying genetic mutation • Measuring endogenous insulin production to determine if insulin needed.
Managing diabetes in pancreatectomized individuals	• Diagnosing and treating insulin-deficient diabetes in these individuals early on • Ensuring life-long insulin and clearly aware of diagnosis • Managing concurrent exocrine failure • Loss of glucagon may also contribute to problematic hypoglycemia • Ensuring access to appropriate diabetes technologies e.g. insulin pumps and continuous glucose monitoring
Impaired hypoglycemia awareness	• Checking individuals know the symptoms of hypoglycemia • Assessing awareness of hypoglycemia using validated scores e.g. Clarke or GOLD score. • Considering adjunctive use of monitoring technologies such as real-time continuous glucose monitoring in those with hypoglycemia unawareness.
No mutation identified (in addition to above)	
Exploring a genetic diagnosis	• Ensuring panel of all genes tested • Undertaking whole exome or whole genome sequencing studies to identify novel genes • Re-characterizing type of hyperinsulinism and considering alternative diagnosis
Counseling around diagnostic uncertainty	• Ensure adequately knowledgeable about their condition • Symptom control • Need to continue medical therapy • Pregnancy

### Developing the HH transition pathways

**Fig. 3:**
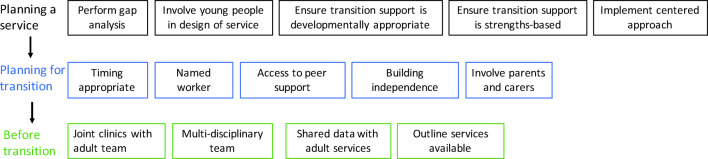
Flow chart of the stages in planning transition. Various factors should be considered before the young person is actually transitioned to adult services. The timing of transition is critical and should be individualized according to the assessment of the multi-disciplinary team. Box 1 Quotes from patients, carers and other family members on the ideal features of a transition clinic. Seeing older patients in clinic waiting areas who might be in the advanced stages of the same condition is scary. Understanding that there are other conditions in the same clinic, or that treatments have changed, helps to remove some of the fear. Young people are often used to being told off and will sometimes try to avoid this by simply not going to an appointment if they are running late. Knowing who they can contact can help prevent this. Will we see one of a team or a named Consultant? I’d like to see the same person for the first few appointments so that we can establish a good relationship.

In line with NICE transition service guidelines [178], the optimal pathway for transitioning individuals with HH must be developed, summarized in Fig. 3. This includes:

- *Planning a service:* Young people should be involved in designing the service.
- *Planning for transition,* which should be appropriately timed for the individual.
- *Before Transition:* young adults should have joint clinics with the pediatric and adult teams.

## Concluding remarks and future directions

HH is a challenging condition to treat due to its heterogeneity. Despite diagnostic and therapeutic advances, HH remains an important cause of morbidity in children, still accounting for 26–44% of permanent intellectual disabilities, especially in neonatal-onset patients. The increasing use of NGS target panels, combined with clinical, biochemical and imaging findings allows differentiating the diagnostic management of children with F-CHH, surgically curable, from those with D-CHH, more conservatively treated with pharmacological and nutritional interventions [200].

There is now more research of use of CGM in children with HH which might help to detect early hypoglycemia leading to prompt management [93]. There have been reports of pitfalls of 18F-DOPA PET-CT in accurately detecting focal lesions in CHH [106, 173]. Glucagon-like-peptide-1 (GLP-1) receptor analogs are the latest agents being used in the detection of insulinomas in adults [108, 109] and are similarly being trialed in children to detect F-CHH.

Over the last few years, numerous medications have been tried in children with HH [201]. For instance long-acting somatostatin analogues and mTOR inhibitors (sirolimus) have been used in various groups of children with varied response [133–136, 140–142, 148]. There is potential use of insulin receptor antagonists as a therapeutic approach to control hypoglycemia in CHH [202, 203]. Also, reports have documented the use of a stable form of glucagon in adults with hypoglycemia [159, 160], however more clinical trials are required to prove its efficacy in children with HH.

Besides the development of new diagnostic tools and therapeutic agents, clinicians need to become involved in creating/potentiating HH transition clinics to provide optimal ongoing care into adulthood for these patients.
